# Deciphering the role of adjuvant therapy in melanoma and its actual benefits

**DOI:** 10.1111/1346-8138.17093

**Published:** 2024-01-11

**Authors:** Satoshi Fukushima, Azusa Miyashita, Toshihiro Kimura, Haruka Kuriyama, Satoru Mizuhashi, Yuki Ichigozaki, Shinichi Masuguchi

**Affiliations:** ^1^ Department of Dermatology and Plastic Surgery, Faculty of Life Sciences Kumamoto University Kumamoto Japan

**Keywords:** adjuvant, melanoma, OS, real‐world, RFS

## Abstract

Numerous clinical trials have demonstrated a significant improvement in recurrence‐free survival among melanoma patients receiving high‐dose interferon‐α, immune checkpoint inhibitors (pembrolizumab, nivolumab), and BRAF/MEK inhibitors (dabrafenib‐trametinib). This study aimed to investigate whether these findings hold true in real‐world conditions for patients with stage III and IV melanoma. In particular, the study explores the efficacy and side effects of adjuvant therapies, focusing on anti‐PD‐1 antibodies and BRAF/MEK inhibitors. While clinical trials have shown comparable efficacy, differences in side‐effect profiles, especially the persistence of immune‐related adverse events with anti‐PD‐1 antibodies, highlight the need for careful consideration in adjuvant settings. In the absence of established biomarkers for guiding adjuvant therapy decisions, it becomes imperative to transparently communicate the advantages and disadvantages of drug administration to patients. The study also delved into the impact of melanoma subtype and BRAF mutation status on the effectiveness of adjuvant therapy, emphasizing the need for further investigation.

## 1 INTRODUCTION

Decades of clinical trials have been dedicated to studying adjuvant therapy for melanoma, encompassing interventions such as high‐dose interferon‐α, immune checkpoint inhibitors (pembrolizumab, nivolumab), targeted therapy (dabrafenib‐trametinib for BRAF mutant melanoma), and chemotherapy. Notably, these trials have reported a marked improvement in recurrence‐free survival (RFS) among melanoma patients treated with high‐dose interferon‐α, immune checkpoint inhibitors (pembrolizumab, nivolumab), and BRAF/MEK inhibitors.^1^, ^2^, ^3^, ^4^ The use of high‐dose interferon‐α has diminished because of a disproportionate level of toxicity relative to its benefits. This has led to the administration of anti‐PD‐1 antibodies in patients with wild‐type BRAF. Recognizing that clinical trial data often involve selected populations, an analysis of real‐world data becomes essential to address the fundamental question, “Is adjuvant therapy genuinely beneficial in melanoma?” This article reviews recent publications investigating adjuvant therapies for stage III and IV melanoma patients under real‐world conditions. Providing a foundation, we present a summary of long‐term data derived from clinical trials, aiming to discern the effectiveness of anti‐PD‐1 antibodies or BRAF/MEK inhibitors in preventing recurrence and prolonging survival in melanoma patients.

## 2 SUMMARY OF CLINICAL TRIAL DATA

### 2.1 Recurrence‐free survival

#### 2.1.1 Stage III

**FIGURE 1:**
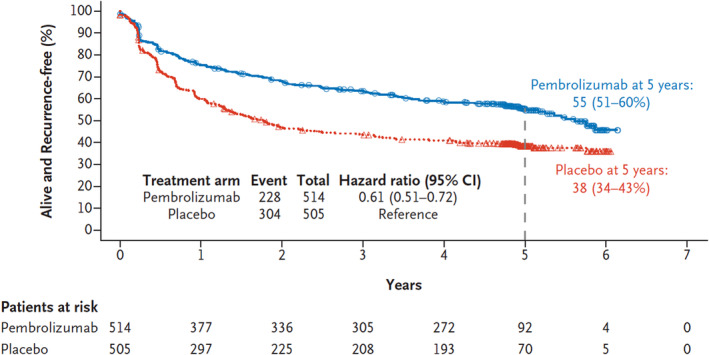
Efficacy outcomes of pembrolizumab. Recurrence‐free survival at 5 years in the pembrolizumab group and the placebo group was 55.4% (95% confidence interval [CI], 50.8–59.8) and 38.3% (95% CI, 33.9–42.7) respectively; the hazard ratio for recurrence or death was 0.61 (95% CI, 0.51–0.72) The positive treatment effect was seen both for locoregional recurrences and distant metastases. Taken from Alexander et al.^5^

In the context of adjuvant therapy for high‐risk stage III melanoma, the administration of 200 mg of pembrolizumab every 3 weeks for up to 1 year, as demonstrated in the KEYNOTE‐054 trial, resulted in significantly extended RFS compared to a placebo.^1^ The most recent long‐term prognosis analysis, encompassing 5‐year follow‐up data on RFS and Progression/Recurrence‐Free Survival 2 (PRFS2), was published in 2022.^5^ In the overall intention‐to‐treat (ITT) population, pembrolizumab continued to show a prolonged RFS compared to placebo, with a 5‐year rate of RFS at 55.4% (95% confidence interval [CI], 50.8–59.8) vs 38.3% (95% CI, 33.9–42.7) and a hazard ratio [HR] for recurrence or death of 0.61 (95% CI, 0.51–0.72) (Figure 1).^5^ Additionally, pembrolizumab exhibited longer distant metastasis‐free survival (DMFS) with a 5‐year rate of 60.6% (95% CI, 56.0–64.9) compared to placebo's 44.5% (95% CI, 39.9–48.9) and a HR for distant metastasis or death of 0.62 (95% CI, 0.52–0.75). The authors concluded that the 5‐year analysis demonstrated sustained improvements in long‐term RFS and DMFS with pembrolizumab compared to placebo in patients with resected stage III melanoma. PRFS2, defined as the time from randomization to the second disease recurrence, progression of the first recurrence, or death, was considered a weaker indicator of overall survival (OS) surrogate markers, positioned between RFS/DMFS and OS. Of the patients, 31.5% in the pembrolizumab group and 43.6% in the placebo group had progression, a second recurrence, or died with a 5‐year rate of PRFS2 at 68.2% (95% CI, 63.9–72.1) in the pembrolizumab group and 55.5% (95% CI, 51.0–59.8) in the placebo group.

In the phase III study CheckMate‐238, adjuvant nivolumab significantly improved RFS compared to ipilimumab in patients with resected stage IIIB–C or stage IV melanoma.^2^ The recent 5‐year update reported efficacy and biomarker findings.^6^ At a minimum follow‐up of 62 months, RFS with nivolumab remained superior to ipilimumab with a HR of 0.72 (95% CI, 0.60–0.86) and 5‐year rates of 50% versus 39%. Five‐year DMFS rates for the nivolumab and ipilimumab groups were 58% and 51%, respectively. Higher levels of tumor mutational burden, tumor PD‐L1, intratumoral CD8 positive T cells, interferon‐γ‐associated gene expression signature, and lower levels of peripheral serum C‐reactive protein were associated with improved RFS and OS with both nivolumab and ipilimumab, albeit with a limited clinically meaningful predictive value.

**FIGURE 2:**
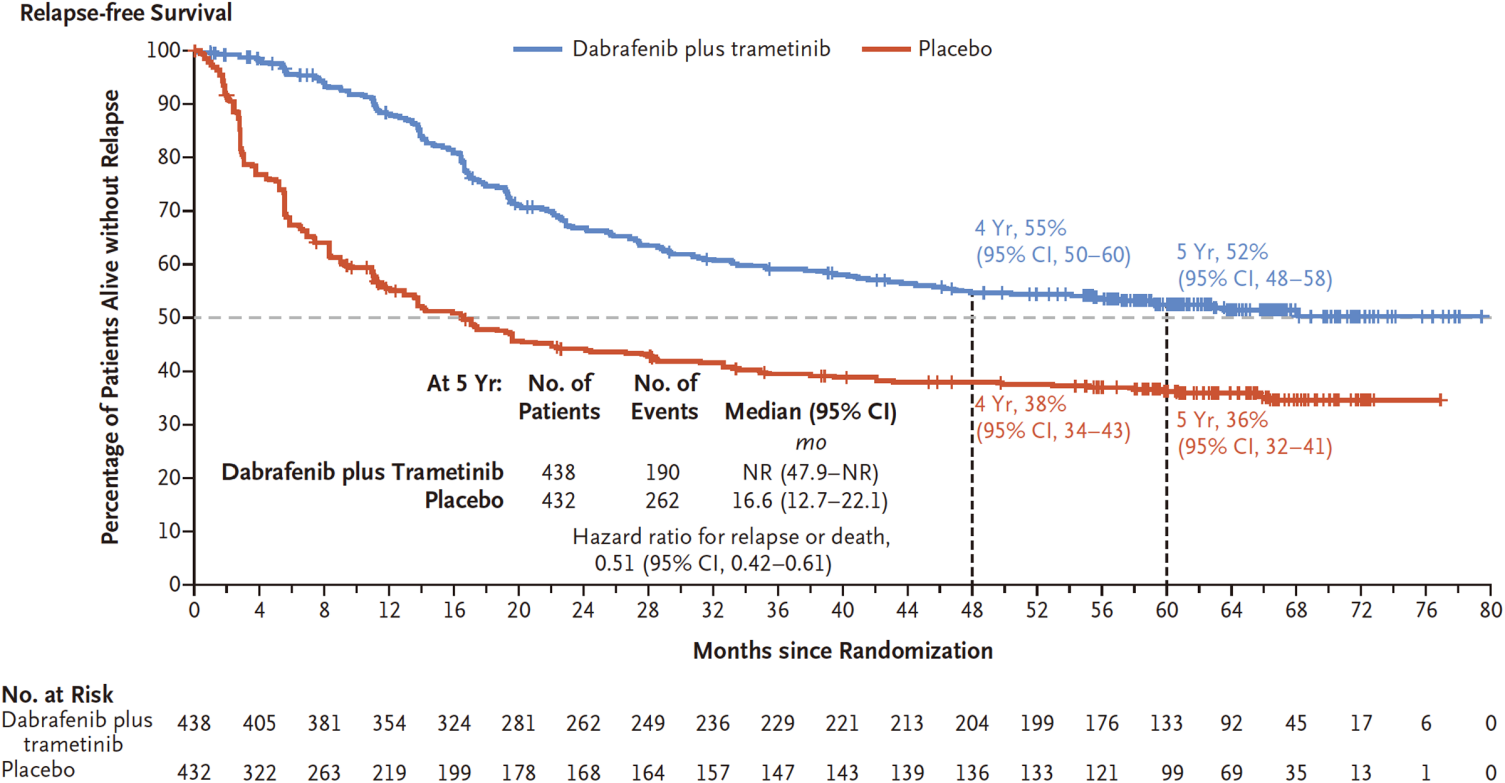
Relapse‐free survival. Shown are Kaplan–Meier estimates of relapse‐free survival in the intention to treat population. The hatch marks indicate censoring of data. AJCC‐7, American Joint Committee on Cancer cancer‐staging manual 7^th^ edition; NR, not reached. Adopted from Dummer et al.^3^

Data on the long‐term prognosis of BRAF/MEK inhibitor (dabrafenib/trametinib) treatment were reported in 2020 (COMBI‐AD).^3^ The minimum follow‐up duration was 59 months for dabrafenib/trametinib and 58 months for placebo. At 5 years, the percentage of patients alive without relapse was 52% (95% CI, 48–58) with dabrafenib/trametinib and 36% (95% CI, 32–41) with placebo, with a HR for relapse or death of 0.51 (95% CI, 0.42–0.61) (Figure 2).^3^ The percentage of patients alive without distant metastasis was 65% (95% CI, 61–71) with dabrafenib/trametinib and 54% (95% CI, 49–60) with placebo, with a HR for distant metastasis or death of 0.55 (95% CI, 0.44–0.70).

#### 2.1.2 Stage II

It has been well known that the prognoses of patients with resected stage IIB/C melanoma and those with resected stage IIIA/B disease are equivalent.^7^ The KEYNOTE‐716 trial evaluated pembrolizumab as adjuvant therapy in patients with completely resected, high‐risk, stage II melanoma.^8^, ^9^ The results of the planned first and second interim analyses for RFS were reported. A total of 976 patients were randomly assigned to receive pembrolizumab (*n* = 487) or placebo (*n* = 489; ITT population). In the second interim analysis (median follow‐up 20.9 months), 72 (15%) patients in the pembrolizumab group and 115 (24%) in the placebo group had a first recurrence or died (HR 0.61; 95% CI, 0·45–0·82). Median RFS was not reached in either group at either assessment timepoint.^8^ At a median follow‐up of 27.4 months (interquartile range, 23.1–31.7), median DMFS was not reached (95% CI not reached [NR]–NR) in either group.^9^ Pembrolizumab significantly improved DMFS (HR 0.64; 95% CI, 0.47–0.88, *p* = 0.0029) versus placebo. The median RFS was 37.2 months (95% CI, NR–NR) in the pembrolizumab group and not reached in the placebo group (95% CI, NR–NR). The risk of recurrence remained lower with pembrolizumab than with placebo (HR 0.64; 95% CI, 0.50–0.84). In 2021, based on the results of this interim analysis, the US Food and Drug Administration (FDA) approved pembrolizumab for the adjuvant treatment of adult and pediatric (≥12 years) patients with stage IIB or IIC melanoma following complete resection.

The phase III, double‐blind, CheckMate‐76K trial assessed 790 patients with resected stage IIB/C melanoma. The patients were randomized 2:1 to receive nivolumab (480 mg) or placebo every 4 weeks for 12 months.^10^ At 7.8 months of minimum follow‐up, nivolumab significantly improved RFS versus placebo, with a HR of 0.42 (95% CI, 0.30–0.59; *p* < 0.0001), with 12‐month RFS of 89.0% versus 79.4% and benefit observed across subgroups; DMFS was also improved (HR 0.47; 95% CI, 0.30–0.72). In October 2023, the FDA approved nivolumab for adjuvant treatment of completely resected stage IIB or IIC melanoma.

To date, the efficacy of BRAF/MEK inhibitors in patients with stage II melanoma has not been reported. The EORTC‐2139/COLUMBUS AD study (NCT05270044) is currently underway, involving 815 patients with resected stage IIB and IIC BRAFV600 mutated melanoma randomized to receive 12 months of adjuvant therapy with encorafenib plus binimetinib or placebo.

### 2.2 Overall survival

To date, ipilimumab is the only adjuvant therapy that has demonstrated a prolongation of OS compared with a placebo in patients with high‐risk resected stage III melanoma.^11^ This does not mean that ipilimumab is superior to anti‐PD‐1 antibodies as adjuvant therapy. Ipilimumab and anti‐PD‐1 antibodies differed in the context in which the clinical trials were conducted. OS is greatly influenced by second‐line treatment, so it is not possible to compare the OS of anti‐PD‐1 antibodies conducted in an era of increased treatment options at relapse with the trials of ipilimumab. Furthermore, this phase 3 trial evaluated ipilimumab at a dose of 10 mg per kilogram in patients with stage III melanoma.^11^ Today, ipilimumab at a dose of 10 mg per kilogram is not used for toxicity reasons and is used at 3 mg for advanced melanoma.^12^ There is a lack of published data on the OS of anti‐PD‐1 antibodies in the adjuvant setting. Consequently, there are no findings from randomized controlled studies to ascertain whether adjuvant anti‐PD‐1 therapy enhances the OS of melanoma patients. Nevertheless, it is reasonable to hypothesize that adjuvant anti‐PD‐1 therapies may exhibit superior efficacy. The final analysis of OS in the KEYNOTE‐054 trial will be conducted after 380 deaths have occurred, a process that may extend beyond the initially planned duration due to the number of events.^1^ In the CheckMate‐238 trial, the five‐year OS rates were reported as 76% for nivolumab and 72% for ipilimumab. However, the data maturity was at 75% (228 out of the 302 planned events).^6^ The results of the impending final analysis are eagerly awaited.

Although presented as an interim analysis, the assessment of OS for BRAF/MEK inhibitors (dabrafenib/trametinib) was reported in 2017.^13^ The 3‐year OS rate was 86% in the combination‐therapy group and 77% in the placebo group (HR for death, 0.57; 95% CI, 0.42–0.79; *p* = 0.0006). However, this level of improvement did not surpass the prespecified interim analysis boundary of *p* = 0.000019. This outcome, being an interim analysis, will undergo a significant difference test in the future. If the same HR and 95% CI are sustained, a significant difference is anticipated.

In conclusion, no randomized controlled data are available to address the question of whether adjuvant therapy improves OS. Recently, Eljilany et al. provided a comprehensive summary of RFS and OS in each clinical trial in a review article on adjuvant therapy.^14^

## 3 REAL WORLD EVIDENCE RELAPSE‐FREE SURVIVAL AND OVERALL SURVIVAL

Ascierto et al. reported on 611 patients with stage III and IV resected melanoma enrolled in an Italian Expanded Access Program (EAP) receiving nivolumab.^15^ After a median follow‐up of 23 months, the RFS in the ITT population was 76.6% at 1 year and 59.6% at 2 years. The 1‐ and 2‐year DMFS rates were 83.7% and 71.2%, respectively. The OS rate in the ITT population was 93.8% at 1 year and 85.5% at 2 years. No significant differences in RFS were observed based on BRAF status.

Rogiers et al. conducted a real‐world, observational study evaluating the effectiveness and safety of adjuvant nivolumab in patients with completely resected stage III or stage IV melanoma in clinical practice in Belgium and Luxembourg (PRESERV MEL).^16^ The study included 152 patients, and at a minimum follow‐up of 11.4 months, the 12‐month and 18‐month RFS rates were 74.7% and 68.4%, respectively, with a median RFS not reached. Cancer‐specific, disease‐specific, and general health‐related quality of life were maintained during and after treatment. The authors concluded that nivolumab demonstrates real‐world efficacy and safety as an adjuvant treatment for patients with completely resected stage III or IV melanoma.^16^

Rigo et al. reported real‐world data primarily from Stage III patients treated with adjuvant anti‐PD‐1 antibodies or BRAF/MEK inhibitors during a median observation period of 22.4 months.^17^ A total of 149 patients (49.7% with BRAF V600E/K genotypes) were included. Anti‐PD‐1 antibodies were administered to 86.5% of the patients, while 13.4% received BRAF/MEK inhibitors. The recurrence rate was 31.5%, with 1‐year and 2‐year RFS rates of 79% and 62%, respectively. Similar recurrence rates were observed with anti‐PD‐1 antibodies and BRAF/MEK inhibitors. Long‐term toxicity affected 27.4% of the patients, with common endocrinopathies and late‐emergent immune‐related adverse events. This study replicated the findings of the CheckMate238 trial, confirming the efficacy of nivolumab as an adjuvant treatment for patients with stage III/IV disease. However, the 2‐year RFS rate of 59.6% suggests that approximately 40% of patients experience a relapse within 2 years of surgery.

**FIGURE 3:**
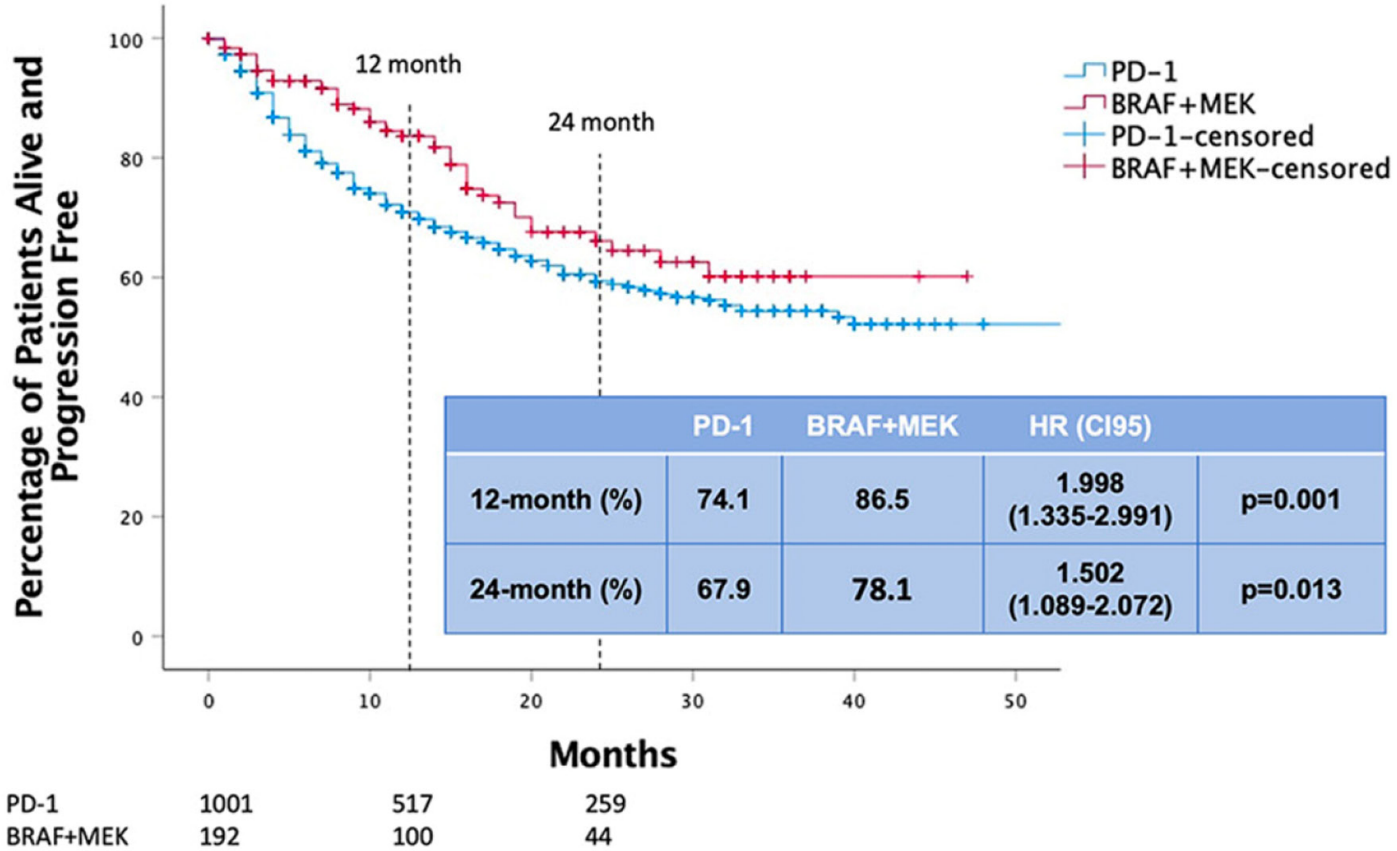
Recurrence‐free survival of patients receiving BRAF/MEK inhibitors (red), and anti‐PD‐1 antibodies (blue). Adopted from Rigo et al.^17^

Recently, Schumann et al. presented real‐world evidence supporting the utilization of anti‐PD‐1 antibodies and BRAF/MEK inhibitors for postoperative adjuvant melanoma across 39 centers in Germany, Austria, and Switzerland.^18^ In their retrospective analysis of 664 cases of nivolumab, 339 cases of pembrolizumab, and 195 cases of dabrafenib/trametinib as postoperative adjuvant therapy, regardless of BRAF mutation, significant findings emerged. The BRAF/MEK inhibitor group exhibited significantly prolonged RFS at 12 and 24 months, although there was no notable difference in OS (Figure 3).^18^ While a direct comparison between the anti‐PD‐1 antibody and BRAF/MEK inhibitor groups specifically for BRAF‐positive cases was not conducted, an evaluation of RFS and OS in the anti‐PD‐1 antibody group, with and without BRAF mutations, revealed that BRAF‐negative cases experienced significantly longer progression‐free survival at 12 months, with no significant distinctions in RFS or OS at 24 months. Notably, patients treated with anti‐PD‐1 antibodies who developed immune‐related adverse events (irAEs) demonstrated lower recurrence rates than those without irAEs (HR, 0.578; 95% CI 0.443–0.754; *p* = 0.001). Comparisons between anti‐PD‐1 and BRAF/MEK inhibitor‐treated cohorts showed no differences in 12‐month RFS and 12‐month OS compared to patients undergoing total lymph node dissection versus sentinel lymph node biopsy only (*p* > 0.05).

An intriguing aspect of Schumann et al.'s report is that 83.7% of patients received anti‐PD‐1 antibodies as adjuvant therapy.^18^ From the patient characteristics data, it is evident that 64.4% of patients with the BRAF‐V600E mutation opted for anti‐PD‐1 antibodies. In contrast, in Japanese clinical practice, most patients with BRAF mutations tend to choose BRAF/MEK inhibitors over immune checkpoint inhibitors because of considerations of efficacy and persistent side effects.^19^ This preference may be influenced by racial differences, melanoma subtypes, and variations in the efficacy of immune checkpoint inhibitors. In Japan, it has been reported that BRAF/MEK inhibitors are equally effective as advanced‐stage treatment compared to Western countries,^20^, ^21^ while immune checkpoint inhibitors are less effective.^22^, ^23^, ^24^ The efficacy and safety profiles of anti‐PD‐1 monotherapy in the adjuvant setting in an Asian population, including a high ratio of acral melanoma, have been reported.^19^ The overall RFS was 60.3% (47/78 cases, 95% CI, 49.2–70.4), with 39.7% of patients (31/78 patients, 95% CI, 29.6–50.8) experiencing relapse during adjuvant PD‐1 antibody treatment. The RFS of acral melanoma was 25.8%, whereas that of high cumulative sun damage was 60.0%, and that of low cumulative sun damage was 57.1%. The acral type had a significantly lower 12‐month RFS rate than the other cutaneous types (*p* = 0.029), appearing to be an independent prognostic factor on multivariate analysis (*p* = 0.015).^19^ Conversely, the efficacy and safety profiles of BRAF/MEK inhibitors in the adjuvant setting were retrospectively analyzed in 36 Japanese BRAF‐mutated patients with advanced melanoma.^25^ The relapse‐free rate (RFR) at 12 months was 82.1% (95% CI, 63.9–92.6). In the 21 patients who completed the protocol, the RFR at 12 months was 85.7% (95% CI, 64.5–95.9). Based on this evidence, it can be inferred that BRAF/MEK inhibitors are commonly used as adjuvant therapies in Japan.

In China, a study explored the relationship between NRAS and tertiary lymphoid structures (TLS) in the adjuvant therapy involving anti‐PD‐1 antibodies for acral melanoma. It observed low efficacy of anti‐PD‐1 in the acral type with an NRAS mutation.^26^ They reported that the efficacy of anti PD‐1 antibodies in the acral type with an NRAS mutation is low. The study involved 21 and 20 patients receiving pembrolizumab and toripalimab, respectively. The median DMFS in the wild‐type NRAS group (*n* = 23) significantly exceeded that in the NRAS mutant group (*n* = 8) (41.1 months vs 9.0 months, *p* < 0.001). Varied levels of TLS infiltration did not significantly impact patient survival. The authors concluded that adjuvant anti‐PD‐1 antibodies may not be suitable for treating acral melanoma with NRAS mutations.^26^ This speculation is interesting, but as it is a study of a small number of cases, the interpretation should carefully be made.

**FIGURE 4:**
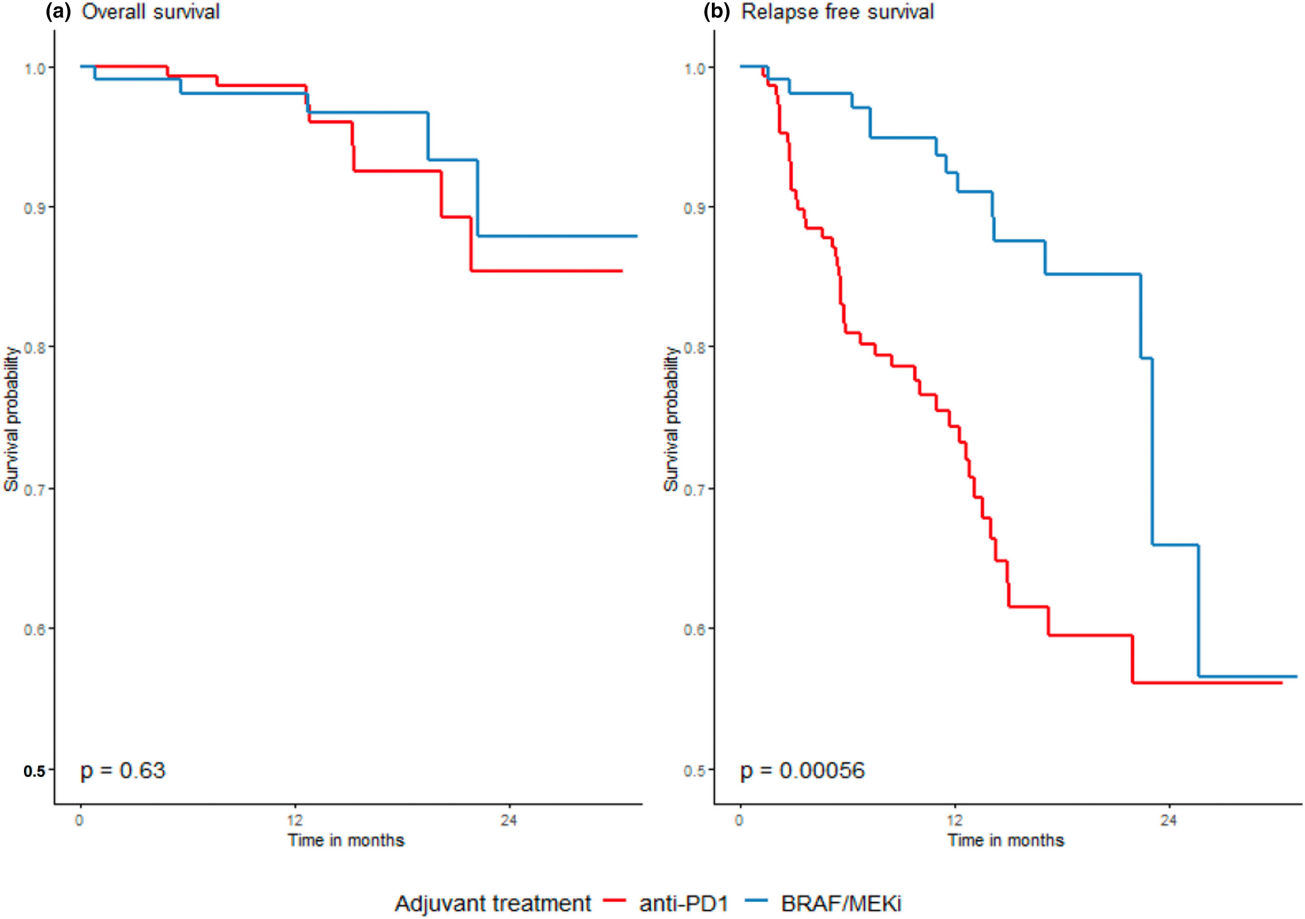
(a) Overall and (b) relapse‐free survival in relation to the type of drugs used; BRAF/MEK inhibitors versus anti‐PD‐1 antibodies. Adopted from Placzke et al.^27^

**FIGURE 5:**
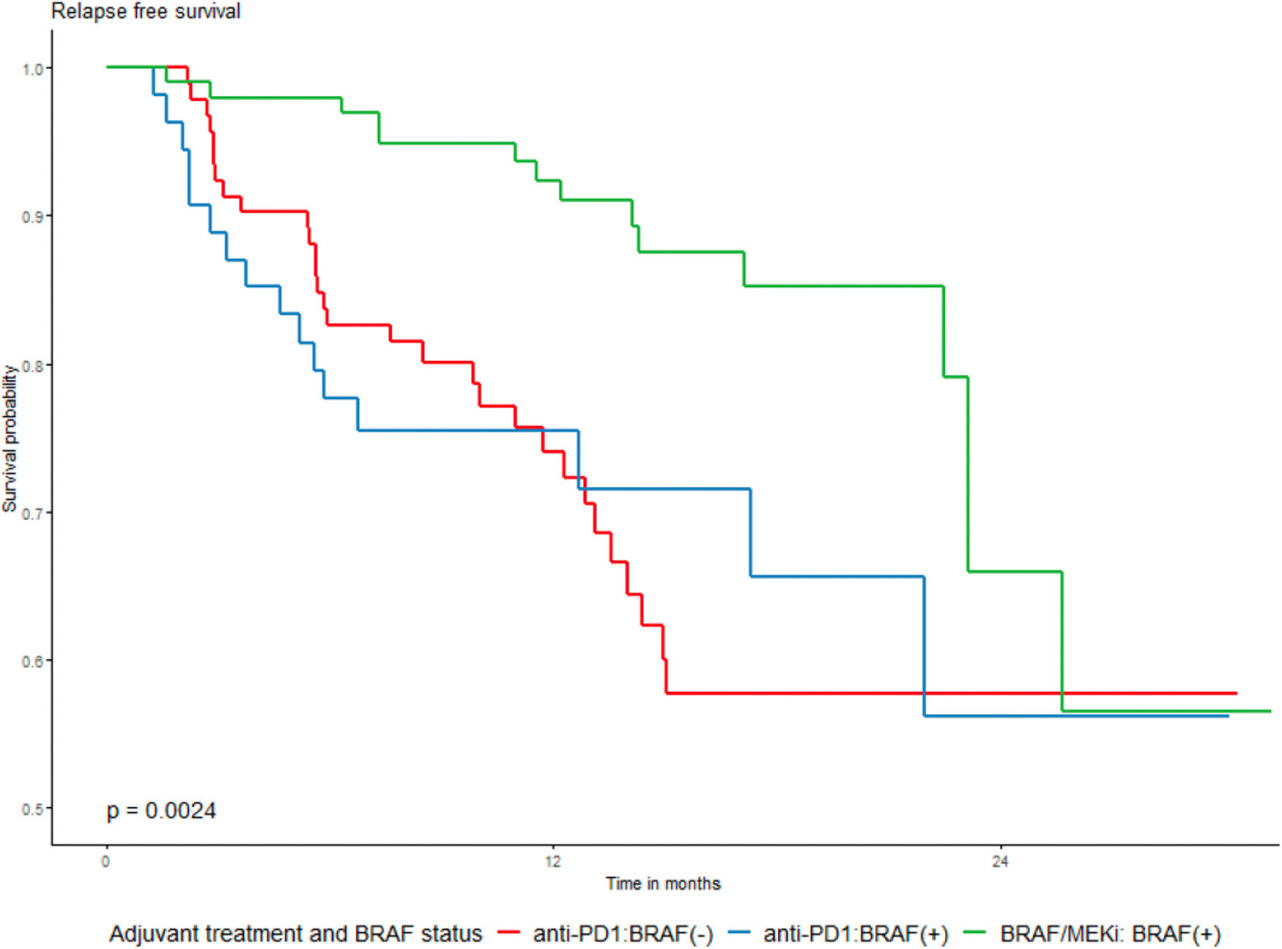
Overall survival by BRAF mutational status and adjuvant drug used; anti‐PD1 or BRAF/MEK inhibitors. Adopted from Placzke et al.^27^

Placzke et al. present real‐world evidence on the use of anti‐PD‐1 antibodies and BRAF/MEK inhibitors for postoperative adjuvant melanoma in eight Polish centers.^27^ A retrospective analysis of 82 cases of nivolumab, 65 cases of pembrolizumab, and 101 cases of BRAF/MEK inhibitors indicated a significantly longer RFS in the BRAF/MEK inhibitor group with no significant difference in OS. The 2‐year OS, RFS, and DMFS rates were 86.7%, 61.4%, and 70.2%, respectively.^27^ Lymph node surgery before adjuvant therapy and the type of drug used did not significantly influence 2‐year OS for the entire group (*p* = 0.6318) (Figure 4a).^27^ However, there was a notably unfavorable difference in RFS rates for anti‐PD‐1 adjuvant treatment, especially early in the observation period (Figure 4b). This disparity persisted in both the overall group and the BRAF/+/group (Figure 5).^27^ BRAF/+/melanoma patients exhibited higher 2‐year RFS rates in the entire cohort and the BRAF/MEK inhibitor‐treated group compared to those treated with anti‐PD‐1 antibodies. The impact of treatment‐related adverse events on OS and RFS was consistent across subpopulations defined by surgery prior to adjuvant treatment.

Lodde et al. report real‐world German data from a DeCOG study with a median observation period of 25.7 months. They assert that BRAF/MEK inhibitors are better in terms of early recurrence and efficacy.^28^ RFS, OS, and melanoma‐specific survival (MSS), and response to subsequent treatment in 589 stage III patients (232 BRAF‐mutated) receiving adjuvant PD‐1 antibodies (*n* = 479) or targeted therapy (*n* = 110) have been reported. The main reasons for premature discontinuation of adjuvant therapy were disease progression in PD‐1 antibodies (28.8%, *n* = 138/479) and adverse events in patients treated with BRAF/MEK inhibitors (28.2%, *n* = 31/110). Among BRAF‐mutated patients, the RFS at 24 months was 49% (95% CI, 40.6–59.0) for PD‐1 antibodies and 67% (95% CI, 58–77) for BRAF/MEK inhibitor‐treated patients. The risk of recurrence was higher for BRAF‐mutated anti‐PD‐1 antibodies group than BRAF/MEK inhibitors group (HR, 1.99; 95% CI 1.34–2.96 vs 2.21; 95% CI 1.48–3.30). The 24‐month MSS was 87% (95% CI, 81.0–94.1) for the anti‐PD‐1 antibodies group and 92% (95% CI, 86.6–97.0) for the BRAF/MEK inhibitors group. Response rates to subsequent systemic treatment for unresectable disease were 22% in the anti‐PD‐1 antibodies group and 16% in the BRAF/MEK inhibitor group.

## 4 ADVERSE EVENTS

In a clinical trial evaluating the combination of dabrafenib and trametinib as adjuvant therapy (COMBI‐AD), the safety analysis included 435 patients in the combination therapy group and 432 patients in the placebo group.^13^ Adverse events were reported in 97% of patients in the combination therapy group and 88% in the placebo group. Among the adverse events occurring in over 10% of patients in the combination therapy group, the most prevalent were pyrexia (63% any grade, 5% grades 3 or 4), fatigue (47% any grade, 4% grades 3 or 4), and nausea (40% any grade, <1% grades 3 or 4). Serious adverse events were observed in 36% of patients in the combination therapy group and 10% in the placebo group. These findings suggest an acceptable side‐effect profile for dabrafenib/trametinib combination therapy as adjuvant treatment. In real‐world scenarios, the side effects of BRAF/MEK inhibitors are generally manageable, with few persistent effects.

Goodman et al. presented the long‐term follow‐up of 318 patients with adjuvant anti PD‐1 antibodies who discontinued treatment due to irAE.^29^ This retrospective, multicenter cohort study analyzed patients treated with adjuvant anti–PD‐1 antibodies in the US and Australia. In this cohort, 63.7% of patients developed acute irAEs during treatment, with 13.8% experiencing grade 3–5 irAEs. During long‐term follow‐up, 36.7% of patients had resolution of chronic irAEs. Among those with persistent irAEs, 59.1% were grade 2 or higher, 44.1% were symptomatic, 25.8% were using therapeutic systemic steroids, and 45.2% were utilizing other management strategies. Common persistent chronic irAEs include hypothyroidism, arthritis, dermatitis, and adrenal insufficiency. Of the 37 patients with chronic irAEs receiving additional immunotherapy, 67.6% experienced no effect, and 32.4% experienced a flare of chronic toxicity. Twenty (54.1%) patients experienced distinct irAEs.^29^

Patients receiving adjuvant therapy included those who did not relapse without treatment. Communicating these benefits accurately to patients is crucial, considering the potential for permanent side effects when using immune checkpoint inhibitors.

## 5 TREATMENT OPTIONS AFTER RECURRENCE

In a retrospective study by Owen et al., characteristics of patients with early relapse and subsequent treatment with adjuvant anti‐PD‐1 antibodies from 16 centers were reported.^30^ Melanoma recurrence occurred in 17% of 850 patients treated with adjuvant anti‐PD‐1 antibodies. The median time to first recurrence from starting adjuvant anti‐PD‐1 antibodies was 4.6 months. Melanoma recurred in most patients during receiving adjuvant anti‐PD‐1 antibodies (*n* = 104, 76%) at a median 3.2 months. Of the 32 patients (*n* = 32; 24%) who experienced recurrence after the cessation of adjuvant anti‐PD‐1 antibodies, the median time to recurrence was 12.5 months. Fifty‐nine (43%) patients experienced locoregional disease only and 77 (57%) had distant disease. Of the patients who experienced locally, 22/59 (37%) experienced distant recurrence. Eighty‐nine (65%) patients received systemic therapy after recurrence. Of the patients who experienced recurrence during adjuvant anti‐PD‐1 antibodies therapy, none (0/6) responded to anti‐PD‐1 antibodies alone, 8/33 (24%) responded to ipilimumab (alone or in combination with anti‐PD‐1 antibodies), and 18/23 (78%) responded to BRAF/MEK inhibitors. Of those who experienced reccurence after discontinuation of adjuvant anti‐PD‐1 antibodies, two out of five (40%) responded to PD1 monotherapy, two out of five (40%) responded to ipilimumab‐based therapy and 9/10 (90%) responded to BRAF/MEK inhibitors.^30^

It seems reasonable that if a patient relapses during treatment with anti‐PD‐1 antibodies, the disease will not respond to treatment with these drugs. Patients who experience relapse during adjuvant therapy should be switched to ipilimumab and nivolumab, or BRAF/MEK inhibitors. The duration of adjuvant therapy with anti‐PD‐1 antibodies monotherapy remains uncertain, and requires further accumulation of information.

## 6 BIOMARKERS IN REAL WORLD DATA

Patients with stage III melanoma, specifically IIIA, IIIB, and IIIC, have different prognoses. The 5‐year RFS rate for stage IIIA disease is 69.4%.^31^ With anti‐PD‐1 antibodies, persistent side effects can occur, emphasizing the need to identify biomarkers for adjuvant therapy recipients. Biomarkers should be easily measurable in hospital settings.

Schumann et al. used a machine‐learning approach to investigate various variables predicting early melanoma recurrence.^18^ The regression model achieved an area under the curve of 0.65, indicating limited predictive capacity. Known coefficients, such as disease stage, tumor thickness, ulceration, lactate dehydrogenase level, and the number of involved lymph nodes at diagnosis, were predictors of disease recurrence. Higher age and absolute leukocyte, eosinophil, and lymphocyte counts were predictors of lower recurrence risk. The addition of other variables did not further improve the model.^18^ Blood‐based dynamic biomarkers, like circulating tumor DNA may help detect minimal residual disease and identify patients benefiting from adjuvant treatment.

## 7 CONCLUSION

This review aimed to address the question of whether adjuvant therapy is genuinely beneficial in melanoma, particularly in terms of prolonging RFS and OS in real‐world scenarios. Current data suggest that anti‐PD‐1 antibodies and BRAF/MEK inhibitors increase RFS, but further information is needed to establish benefits in OS. In the absence of established biomarkers for selecting adjuvant therapy, the advantages and disadvantages of drugs with potential persistent side effects should be clearly explained to patients. The impact of adjuvant therapy may also vary based on melanoma subtype and BRAF mutation status, requiring further investigation. The efficacy of neoadjuvant therapy is also under examination^32^, ^33^ with discussions on appropriate perioperative systemic therapy to be explored in the future.

## CONFLICT OF INTEREST STATEMENT

Satoshi Fukushima has received research, speaking, and/or consulting support from Bristol‐Myers Squibb, Novartis, MSD and Ono. All other authors had no conflicts of interest to declare.
